# Development and validation of nomogram for predicting cognitive frailty with multimorbidity: a cross-sectional study

**DOI:** 10.3389/fpubh.2025.1606505

**Published:** 2025-08-29

**Authors:** Chunbo Guo, Shunyin Liu, Yuehua Liu, Mengxi Zhang, Shan Liu, Liting Zeng, Lu Luo

**Affiliations:** ^1^Clinical Nursing Teaching and Research Section, The Second Xiangya Hospital, Central South University, Changsha, Hunan, China; ^2^Department of Geriatrics, The Second Xiangya Hospital, Central South University, Changsha, Hunan, China

**Keywords:** aged, cognitive frailty, multimorbidity, nomogram, prediction model

## Abstract

**Objectives:**

This study aims to develop and validate a nomogram for cognitive frailty in older patients with multimorbidity.

**Methods:**

From April 2022 to December 1, 2024, a total of 711 older patients participated in the study. The study was conducted at a tertiary hospitals in Changsha, Hunan Province, China. We employed LASSO regression to identify initial variables associated with risk factors for older adults with multimorbidity and subsequently utilized regression analysis to develop predictive models. We collected encompassing demographic information, FRAIL scale scores, Mini-Mental State Examination (MMSE) results, Mini Nutritional Assessment Short Form (MNA-SF) evaluations, Patient Health Questionnaire-9 (PHQ-9) responses, and Athens Insomnia Scale (AIS) ratings. Statistical analyses were performed using R version 4.3.2. The model’s predictive performance was evaluated using receiver operating characteristic (ROC) and area under the curve (AUC). Calibration was assessed via calibration curves, and clinical utility through decision curve analysis (DCA). Internal consistency was validated using bootstrap, and external validity with an independent test dataset.

**Results:**

In this study, the training and validation sets were 498 and 213 patients, respectively. In the training set, there were 183 patients with cognitive frailty with a prevalence of 36.9%. Six initial variables were selected for the LASSO regression, including drinking, constipation, polypharmacy, chronic pain, nutrition, and depression. These six variables were included in the final predictive model. The model demonstrated a concordance index (C-index) of 0.818. Furthermore, AUC for the training and validation sets were determined to be 0.827 and 0.784, underscoring the model’s robust predictive capability.

**Conclusion:**

The high prevalence of cognitive frailty in older patients with multimorbidity should be noted. Efforts to diagnose cognitive frailty and develop targeted interventions in the context of an ageing population and young onset of dementia are of significance in delaying and reversing cognitive frailty.

## 1 Introduction

The global population aged 65 years and older is projected to reach approximately 2.2 billion by 2080 (1). The prevalence of multimorbidity is also increasing as a result of population ageing. Multimorbidity is defined as the coexistence of two or more chronic conditions (2). Currently, the global prevalence of multimorbidity among community-dwelling individuals is estimated to be 37.2% (3). Multimorbidity is significantly associated with higher rates of hospitalisation, increased healthcare expenditure, poorer quality of life,and a higher risk of mortality (4). The consequences also include cognitive decline and frailty (5).

Cognitive frailty (CF), as defined by the International Consensus Group of the International Academy of Nutrition and Ageing (I.A.N.A.) and the International Association of Gerontology and Geriatrics (I.A.G.G.). CF refers to the coexistence of physical frailty and cognitive impairment without other complication such as Alzheimer’s disease or dementia (6). The prevalence of CF in older adults was reported to be 9% (7). In clinical settings, this number ranges from 10.7 to 39.7% (8). People with CF have a higher risk of falls, disability, hospitalization, institutionalization, and mortality (9–11). They are also more likely to develop dementia than those with either physical frailty or cognitive impairment alone (12). Early identification and intervention of CF are essential to mitigate adverse outcomes and support healthy aging.

The evaluation of CF involves assessing both physical frailty and cognitive function. Frailty is usually measured by the Fried frailty phenotype (13), the frailty index (FI) (14) and the FRAIL scale (15). Cognitive function is commonly assessed using the Mini-Mental State Examination (MMSE) (16) and the Montreal Cognitive Assessment (MoCA) (17). However, early identifying CF remains challenging due to the lack of a unified diagnostic framework, even though relatively reliable criteria exist. Additionally, since CF is assessments combine measures of frailty and cognitive impairment, the predictive accuracy of these methods remains limited. These challenges emphasize the need for effective predictive models that include multiple risk factors to enhance early detection and intervention strategies.

Risk prediction models can help identify individuals at high risk of CF, allowing for timely preventive intervention. Traditional statistical models has been widely used to predict frailty and cognitive impairment (18, 19). The Least Absolute Shrinkage and Selection Operator (LASSO) regression is particularly effective because it can handles high-dimensional data, improves model clarity, and enhances predictive accuracy. It selects the most relevant predictors and reduces overfitting (20). Using LASSO regression to predict CF may create a stronger and more practical model.

Several prediction models have been developed to estimate the risk of CF, but most are not designed specifically for patients with multimorbidity (21). Existing models often focus on frailty or cognitive impairment independently. They have overlooked the complex interactions between chronic diseases and their combined effects on CF. Notably, only one prediction model targeted older adults with multimorbidity, but its predictors were not commonly used in community clinics, and its performance in the hospital setting. Furthermore, this model lacks methodological validation of its clinical applicability (22). These limitations highlight the need for more comprehensive, clinically applicable models that clearly reflect the complex nature of CF in people with multimorbidity.

The development a prediction model of CF is a complex and influenced by many factors. It is important to include as many relevant risk factors as possible to reduce bias caused by missing key variables. However, traditional regression methods often have difficulties with small sample sizes and high-dimensional data. To address these challenges, this study aims to develop and validate a new nomogram for predicting CF in patients with multimorbidity. By employing LASSO regression, this methods aims to improve predictive accuracy and clinical usefulness through effective selection of variables.

## 2 Materials and methods

### 2.1 Study sites and population

A cross-sectional survey was conducted from 1 April 2022 to 1 December 2024 in a tertiary-level hospital in Hunan Province, China, which serves as a regional referral center providing specialized and comprehensive medical services. The inclusion criteria were: ① age ≥60 years; ② patients with 2 or more chronic diseases. Patients with critical illness who could not cooperate with the survey were excluded. To develop the predictive model, we followed the Prediction model Risk Of Bias and Applicability of Prediction Model Studies (PROBAST) guidelines, which recommend a minimum of 20 events per variable (EPV) to ensure stable model performance (23). Previous studies have reported a 9% prevalence of CF among older adults (7), so we calculated that at least 223 cases were needed for the modeling group to meet the EPV requirement. To facilitate both model development and validation, we divided the total sample into two groups: 70% for model development and 30% for validation. Therefore, the total sample size was at least 319 cases. This study was approved by the Ethics Committee of the Second Xiangya Hospital of Central South University (2022074).

### 2.2 Data collection

Study data were gathered through face-to-face assessments and self-reported questionnaires. Before conducting the survey, the nurse manager of the geriatric ward was contacted to obtain permission for patient participation. During the recruitment, the nurse manager helped screen patients according to inclusion criteria. To ensure consistency, all investigators received standardized training on clearly explaining the survey’s purpose, content, estimated completion time, and confidentiality measures. After obtaining informed consent, questionnaires were given to participants, which took about 15–20 min per patient. All participants received a standardized verbal introduction explaining the purpose and procedures of the study prior to completing the questionnaire. Investigators provided additional explanations if participants required further clarification. After participants completed the questionnaires, investigators collected and checked them on-site to ensure the accuracy and completeness of data before concluding the data collection process.

### 2.3 Measures

#### 2.3.1 Patient demographic information form

Based on the literature review and clinical experience, the research team designed a patient demographic information form. The form included the following items: gender, age, education, marital status, Body Mass Index (BMI), leg circumference, history of smoking, history of drinking, constipation, eyesight, hearing, polypharmacy. History of falls within 1 year, residence status, social participation, and chronic pain.

#### 2.3.2 Diagnosis of CF

CF was defined as the concurrent presence of pre-frailty or frailty and mild cognitive impairment (6). The FRAIL scale was used to assess frailty, and the MMSE was used to evaluate cognitive function. The FRAIL scale comprises five items—Fatigue, Resistance, Ambulation, Illnesses, and Loss of weight—with each item scored as 0 or 1 (15). A total score of 1–2 indicates pre-frailty, while a score of 3–5 indicates frailty. The MMSE is a 30-point questionnaire that evaluates different cognitive domains, including orientation, registration, attention and calculation, recall, and language (16). Scores ranges from 18 to 23 suggest mild cognitive impairment, and scores below 18 indicate more severe impairment. To account for educational influences on cognitive performance, the following cutoff scores were applied: ≤17 for individuals with no formal education, ≤20 for those with primary education, and ≤24 for those with junior high school education or higher.

#### 2.3.3 Mini nutritional assessment short form (MNA-SF)

MNA-SF was used to evaluate the nutritional status of participants (24). It included 6 items on weight change past 3 months, BMI, stress, mobility, mental illness and diet. The total score ranges from 0 to 14, with scores of 12–14 indicating normal nutrition, 8–11 indicating a risk of malnutrition, and 0–7 indicating malnutrition.

#### 2.3.4 Patient health questionnaire-9 (PHQ-9)

The PHQ-9 was utilized to assess depression of participants. It is a validated self-report instrument with nine items that measure the frequency of depressive symptoms in the past 2 weeks (25). Each item is scored on a Likert 4 scale ranging from 0 (“not at all”) to 3 (“nearly every day”), resulting a total score between 0 and 27. A total score of less than 5 suggests the absence of depressive symptoms, while a score of 5 or higher indicates the presence of depressive symptoms. Higher scores indicate greater severity of depressive symptoms.

#### 2.3.5 Athens insomnia scale (AIS)

The AIS was used to evaluate sleep disturbances of participants (26). AIS comprises eight items covering different aspects of sleep in the past month. AIS include difficulty falling asleep, awakenings during the night, awakening earlier than desired, total sleep duration, overall quality of sleep, daytime well-being, daytime functioning, and daytime sleepiness. Each item is rated on a scale from 0 to 3, with the total score ranges from 0 to 24. A score of 6 or higher suggesting the presence of insomnia. The higher scores indicating more severe insomnia symptoms.

### 2.4 Statistical analysis

Statistical analyses were conducted using R 4.3.2 software. As all continuous variables followed a non-normal distribution, they were summarized using the median and interquartile range *M*(*P*_25_,*P*_75_), while categorical data were presented as counts and percentages (*n*, %). We applied LASSO regression to select the initial variables. The value of the optimal regularization parameter lambda (*λ*) was identify through 10-fold cross-validation. Subsequently, a multivariate logistic regression was employed to develop a predictive model for the risk of CF in older patients with multimorbidity. The predictive performance of the model was evaluated using the receiver operating characteristic (ROC) curve and area under the curve (AUC). Model calibration and clinical utility were assessed through calibration curves and decision curve analysis (DCA). Internal consistency was performed using the bootstrap method, while external validity was conducted with an independent test dataset.

The study data was divided into training set (70%) and validation set (30%). The performance of column line plots was evaluated in both the training and validation sets to assess discrimination and calibration. Normality was tested using the ‘nortest’ package. The ‘CBCgrps’ package was employed to generate the baseline feature table, while LASSO regression was performed using the ‘glmnet’ package to select the initial predictor variables. For model discrimination, ROC curves were constructed using the ‘pROC’ and ‘ggplot2’ packages, with corresponding calculations of AUC. Regression modeling and calibration curve plotting were conducted using the ‘rms’ package. Model performance and cross-validation procedures were implemented via the ‘caret’ package. Additionally, DCA was performed using the ‘rmda’ package to evaluate the clinical utility of the predictive model.

## 3 Results

### 3.1 Demographic characteristics of participants

**Figure 1:**
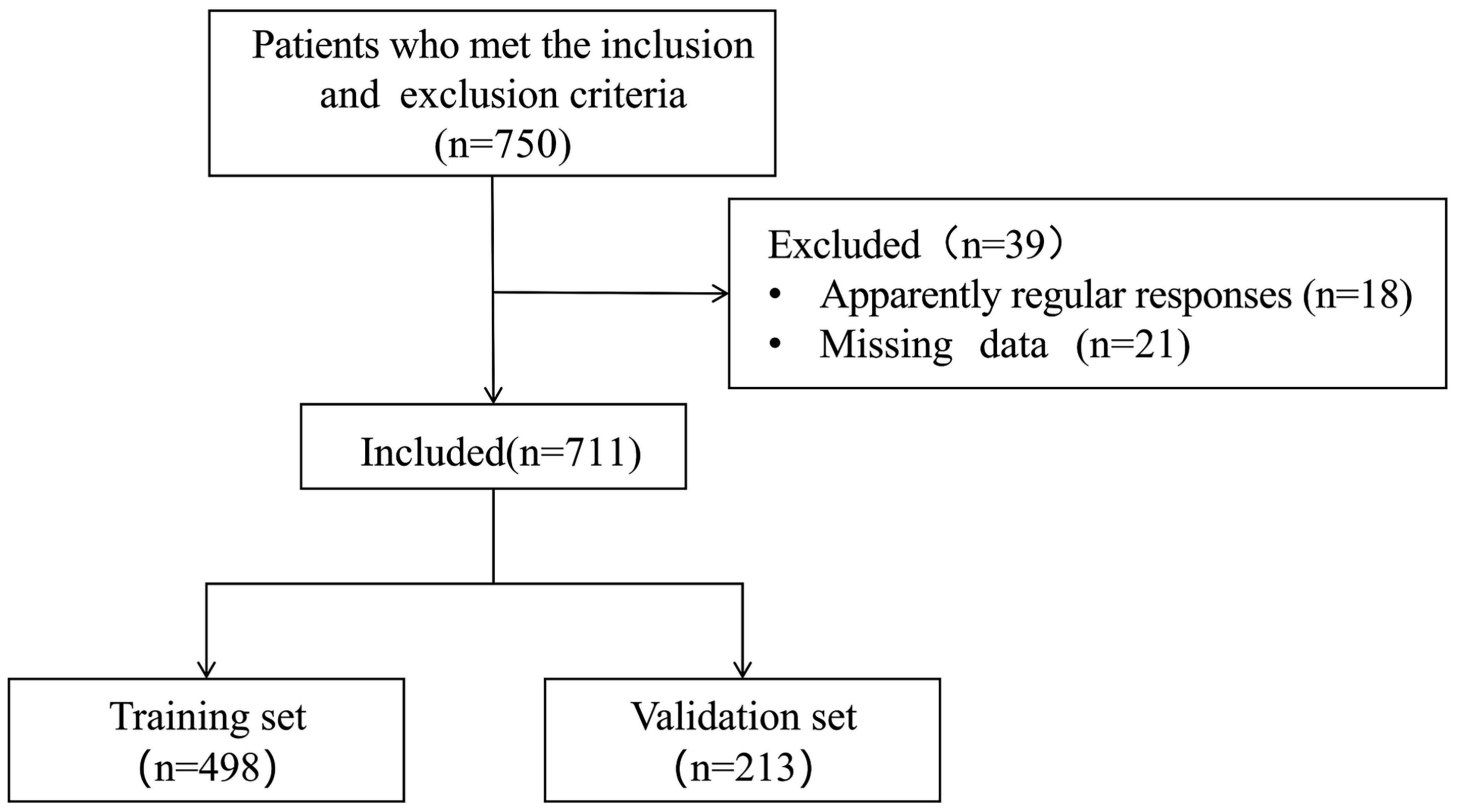
Flow chart of participants.

A total of 750 patients were recruited. However, 18 questionnaires with apparently regular responses and 21 questionnaires with missing data were excluded. The effective response rate was 94.8%. Table 1 presented are descriptive analyses of the general demographic, health behaviour, health history and variable characteristics of the 498 participants in the training set. The recruitment process for study participants is shown in Figure 1.

**Table 1:** Characteristics of CF in older patients with multimorbidity.

Variables[*n* (%)/*M*(*P*_25_,*P*_75_)]	Total (*n* = 498)	Non-CF (*n* = 315)	CF (*n* = 183)
Gender			
Male	245 (49.2)	155 (49.2)	90 (49.2)
Female	253 (50.8)	160 (50.8)	93 (50.8)
Age	77.5 (72, 84)	77 (71, 83.5)	78.5 (74, 84)
Education			
Illiteracy	18 (3.6)	11 (3.5)	7 (3.8)
Primary school	117 (23.5)	77 (24.4)	40 (21.9)
Middle school	99 (19.9)	53 (16.8)	46 (25.1)
High School	124 (24.9)	85 (27.0)	39 (21.3)
University and above	140 (28.1)	89 (28.3)	51 (27.9)
Marital status			
Widowed	135 (27.1)	93 (29.5)	42 (23)
Divorce	4 (0.8)	3 (1)	1 (0.5)
Married	358 (71.9)	219 (69.5)	139 (76)
Single	1 (0.2)	0 (0)	1 (0.5)
BMI	23.55 (21.4, 25.9)	23.4 (21.3, 26.2)	23.6 (21.4, 25.8)
Leg circumference (cm)	32.5 (30, 34.77)	32.3 (30, 34.25)	32 (30.25, 35)
Smoking			
Still smoking	140 (28.1)	82 (26)	58 (31.7)
Quit smoking	104 (20.9)	61 (19.4)	43 (23.5)
Never smoked	254 (51)	172 (54.6)	82 (44.8)
Drinking			
Still drinking	154 (30.9)	71 (22.5)	83 (45.4)
Quit drinking	83 (16.7)	53 (16.8)	30 (16.4)
Never drank	261 (52.4)	191 (60.6)	70 (38.3)
Constipation			
Yes	180 (36.1)	88 (27.9)	92 (50.3)
No	318 (63.9)	227 (72.1)	91 (49.7)
Eyesight			
Problematic and affects daily life	73 (14.7)	60 (19)	13 (7.1)
Problematic but do not affect daily life	253 (50.8)	151 (47.9)	102 (55.7)
No problem	172 (34.5)	104 (33)	68 (37.2)
Hearing			
Problematic and affects daily life	56 (11.2)	43 (13.6)	13 (7.1)
Problematic but do not affect daily life	77 (15.5)	38 (12.1)	39 (21.3)
No problem	365 (73.3)	234 (74.3)	131 (71.6)
Polypharmacy			
Yes	239 (48)	121 (38.4)	118 (64.5)
No	259 (52)	194 (61.6)	65 (35.5)
Falls within a year			
Yes	154 (30.9)	89 (28.3)	65 (35.5)
No	344 (69.1)	226 (71.7)	118 (64.5)
Solitude			
Yes	53 (10.6)	31 (9.8)	22 (12)
No	445 (89.4)	284 (90.2)	161 (88)
Social participation			
Never participate	138 (27.7)	86 (27.3)	52 (28.4)
Occasionally participate	119 (23.9)	67 (21.3)	52 (28.4)
Often participate	122 (24.5)	98 (31.1)	24 (13.1)
Take the initiative to participate and be actively involved.	119 (23.9)	64 (20.3)	55 (30.1)
Chronic pain			
Yes	242 (48.6)	119 (37.8)	123 (67.2)
No	256 (51.4)	196 (62.2)	60 (32.8)
Nutrition			
Malnutrition	50 (10)	8 (2.5)	42 (23)
Nutritional risk	169 (33.9)	91 (28.9)	78 (42.6)
Normal	279 (56)	216 (68.6)	63 (34.4)
Depression			
Yes	113 (22.7)	44 (14)	69 (37.7)
No	385 (77.3)	271 (86)	114 (62.3)
Insomnia			
Yes	196 (39.4)	103 (32.7)	93 (50.8)
No	302 (60.6)	212 (67.3)	90 (49.2)

BMI, body mass index.

### 3.2 Variable selection based on lasso regression

**Figure 2:**
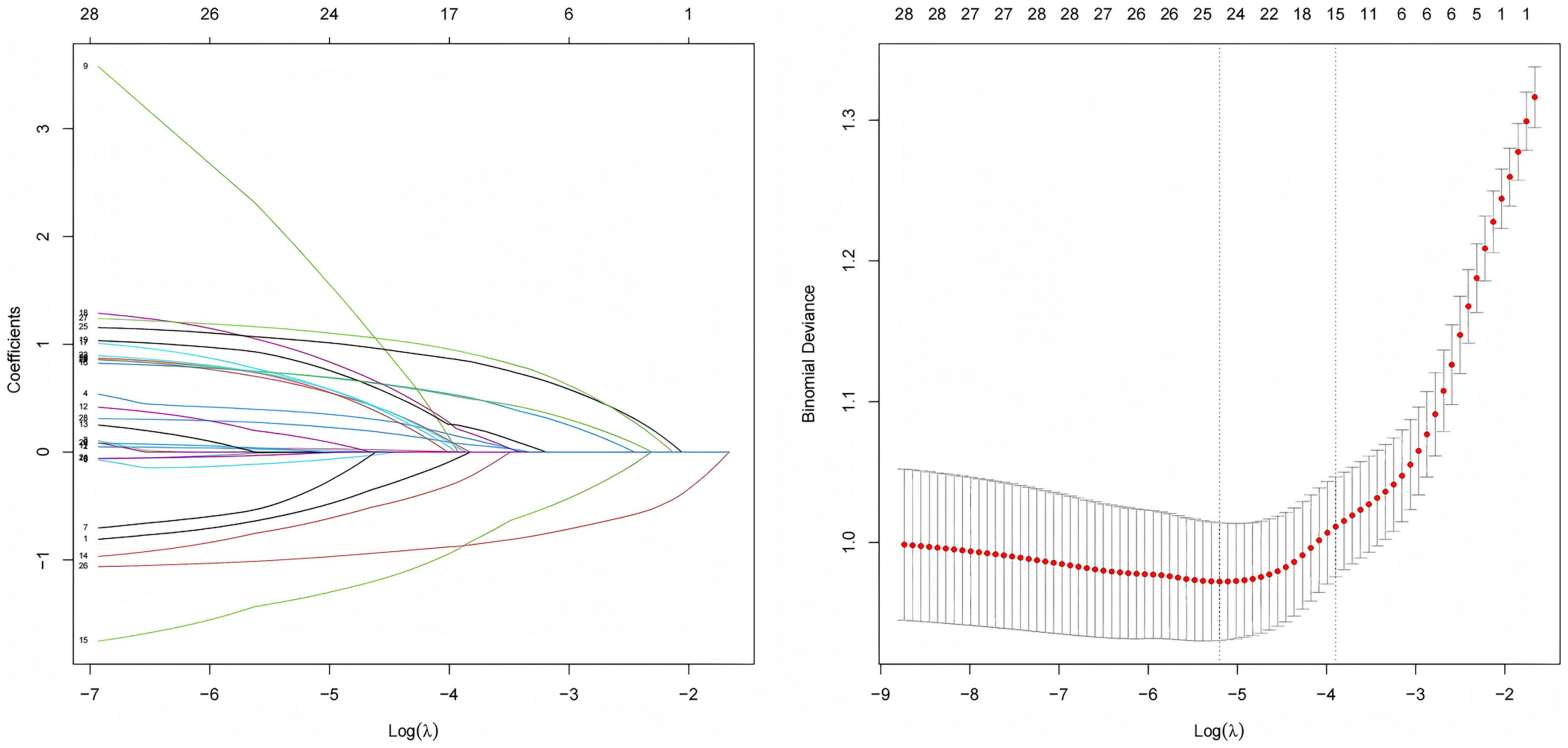
Results of the LASSO regression.

CF was used as the dependent variable, with negative cases assigned a value of 0 and positive cases assigned a value of 1. LASSO regression was employed to compress the coefficients of the independent variables by adjusting the penalty coefficient (*λ*). As shown in Figure 2, the process continued until the coefficients of some independent variables were reduced to zero. The optimal *λ* was chosen using cross-validation, and the mean squared error of log (λ) was plotted to show the selection process. The optimal λ was identified as 0.056, resulting in the selection of six predictor variables, including drinking, constipation, polypharmacy, chronic pain, nutrition, and depression.

### 3.3 Development of the nomogram for CF among patients on multimorbidity

A predictive model was developed using logistic regression analysis, with CF as the dependent variable and independent variables selected through LASSO regression analysis. The final prediction model incorporated six key variables, as detailed in Table 2.

**Table 2:** Results of logistic regression analysis.

Variables	Coefficient	SE	Wald	*p*	OR	95%CI
Drinking						
Still drinking					1 (Ref)	
Quit drinking	−0.883	0.338	6.808	0.009	0.414	0.213–0.803
Never drank	−1.412	0.265	28.372	<0.001	0.244	0.145–0.410
Constipation	0.763	0.241	10.047	0.002	2.144	1.338–3.436
Polypharmacy	0.747	0.229		<0.001	2.111	1.347–3.310
Chronic pain	1.152	0.233	24.521	<0.001	3.165	2.006–4.993
Nutrition						
Malnutrition					1 (Ref)	
Nutritional risk	−1.526	0.473	10.423	0.001	0.217	0.086–0.549
Normal	−2.469	0.467	27.902	<0.001	0.084	0.034–0.212
Depression	1.285	0.262	24.060	<0.001	3.614	2.163–6.038

SE, standard error; 95%CI, 95%Confidence interval.

**Figure 3:**
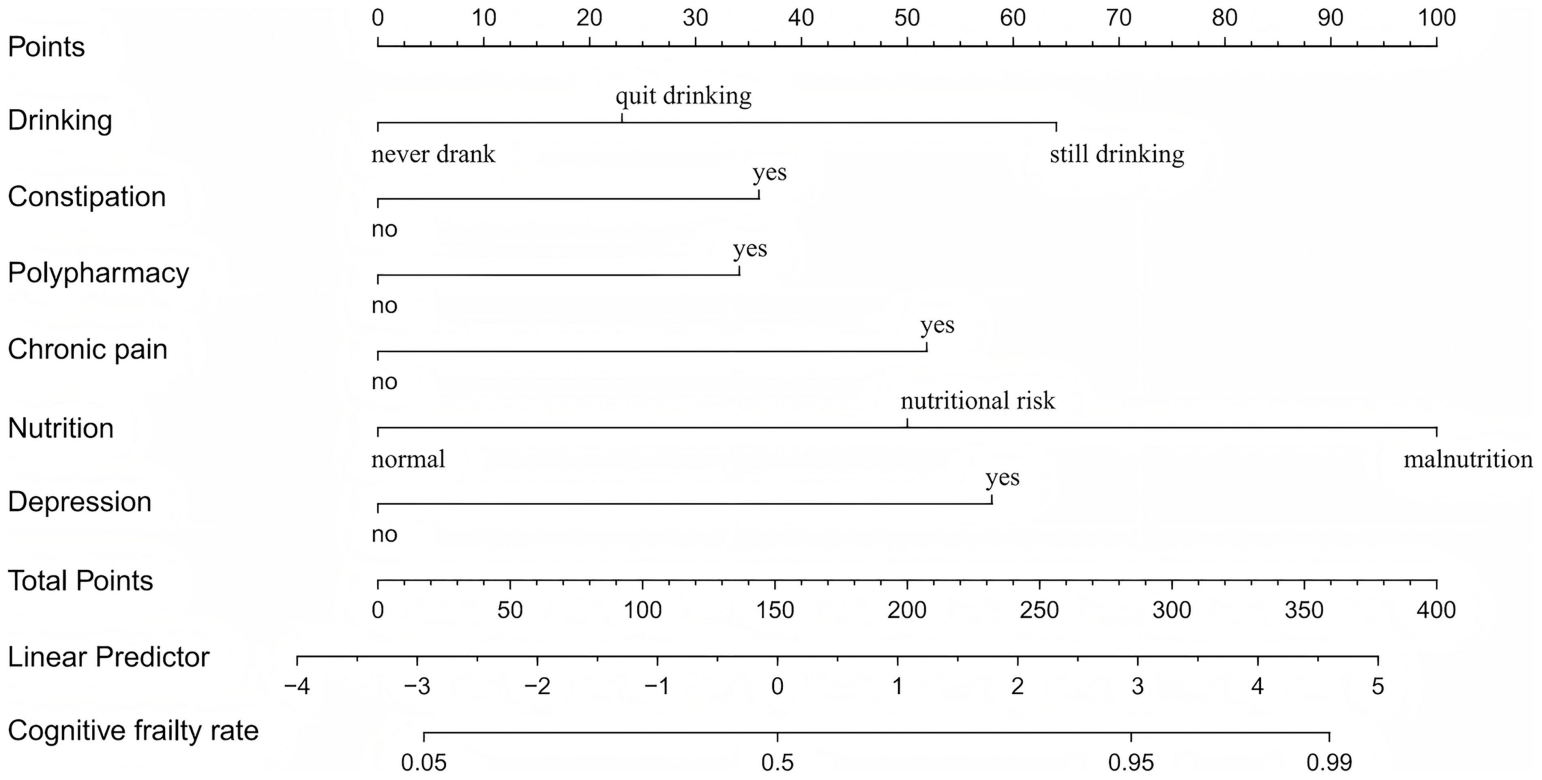
Nomogram for predicting risk of CF.

As shown in Figure 3, based on the logistic regression results, a nomogram was constructed to assess CF in older patients with multimorbidity. Each variable corresponds to a specific point value, and the total score is derived by summing the points of all selected variables. This total score indicates the CF risk of individual.

### 3.4 Evaluation of the nomogram model

**Figure 4:**
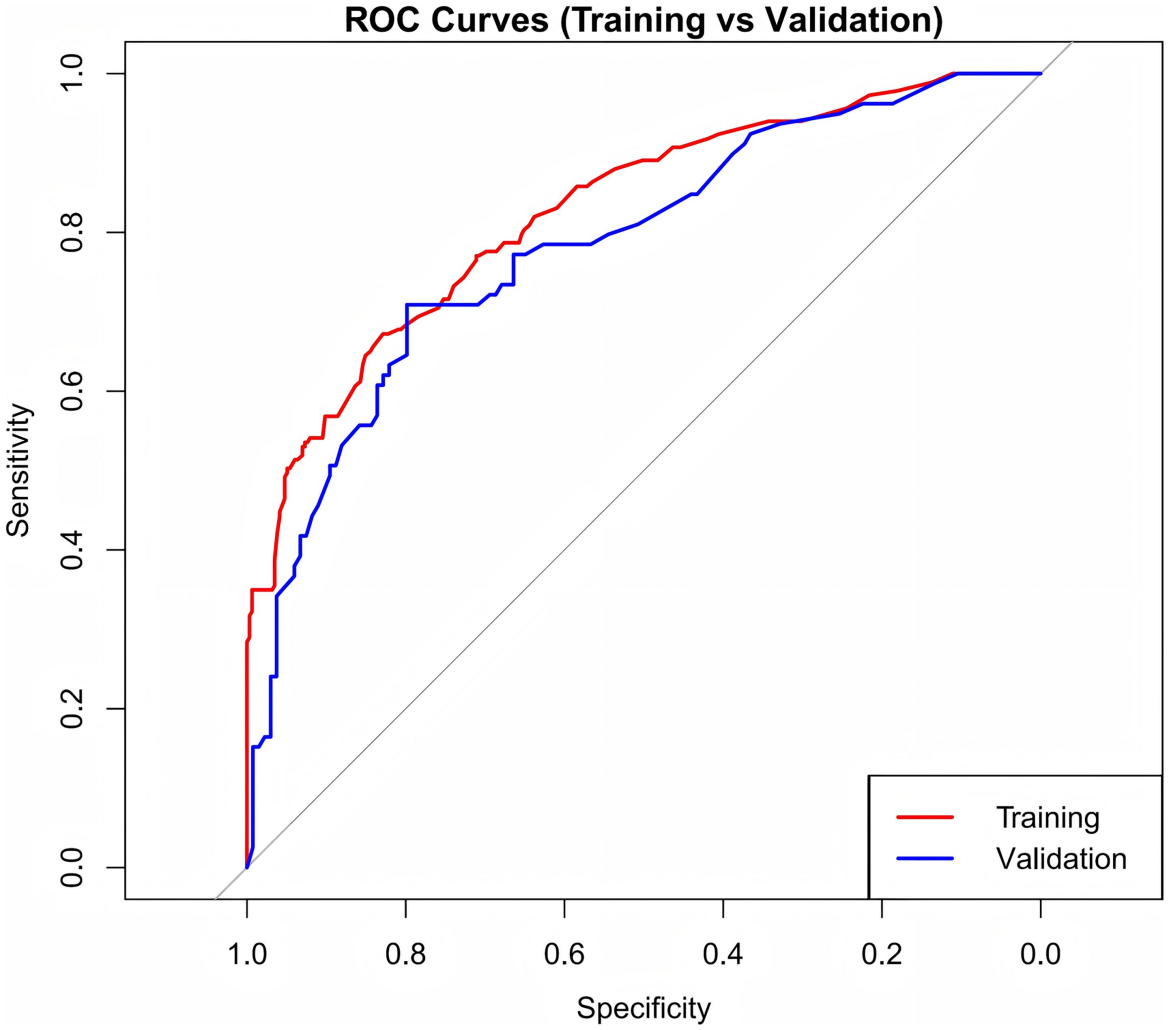
ROC of the predictive nomogram.

Internal validation was conducted using the bootstrap method, resulting in a concordance index (C-index) of 0.818, demonstrating robust model performance. As shown in Figure 4, the AUC values were 0.827 for the training set and 0.784 for the validation set. These results suggest that the model exhibits strong ability in distinguishing patients with CF from those without.

**Figure 5:**
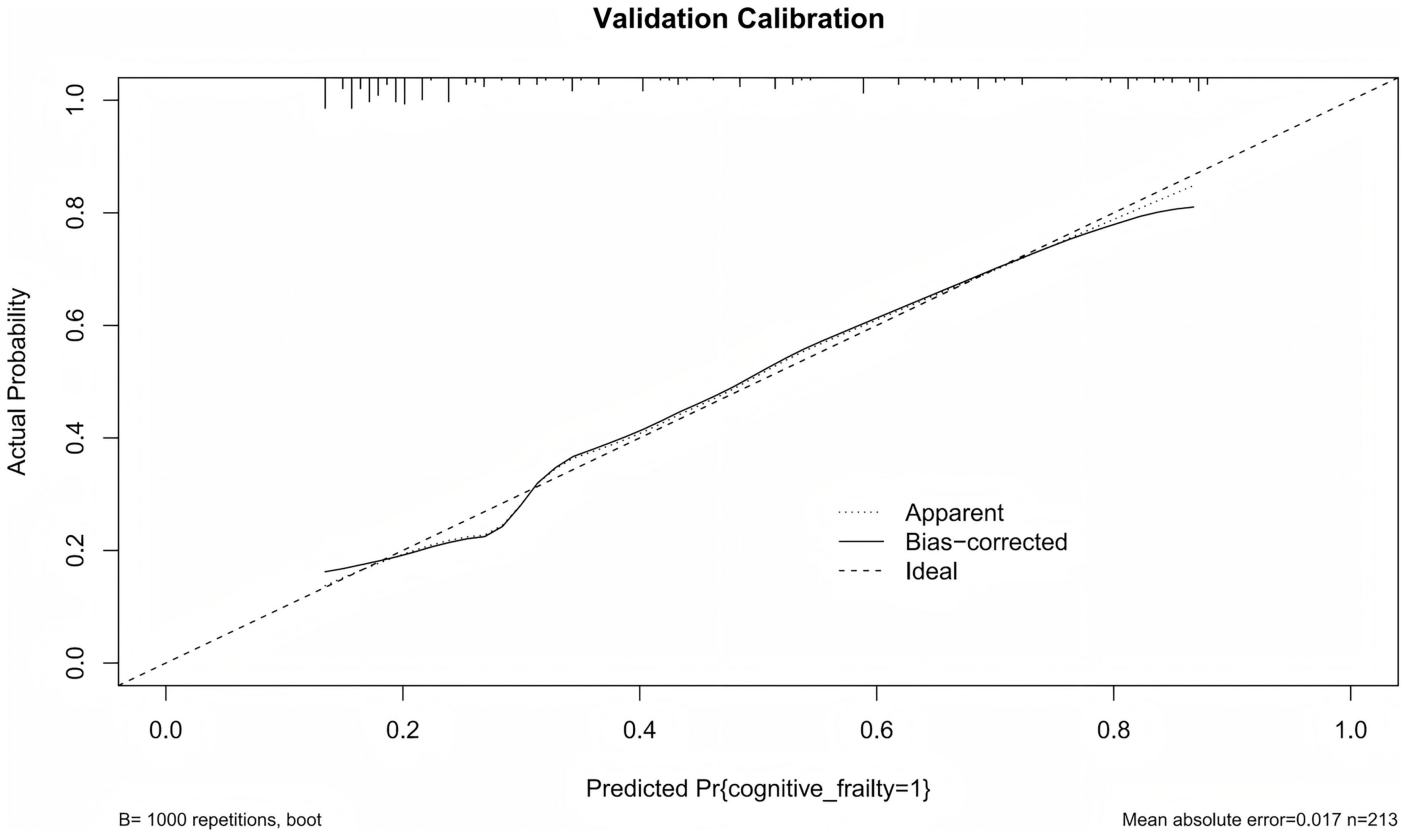
Calibration curve of the predictive nomogram.

Calibration curves were used to evaluate the agreement between the predicted probabilities from the model and the observed probabilities. As shown in Figure 5, the actual calibration curve closely follows the ideal diagonal line. This indicats that the predicted probability of CF in older patients with multimorbidity is highly consistent with the observed probability. This results suggests that the model demonstrates good calibration performance.

**Figure 6:**
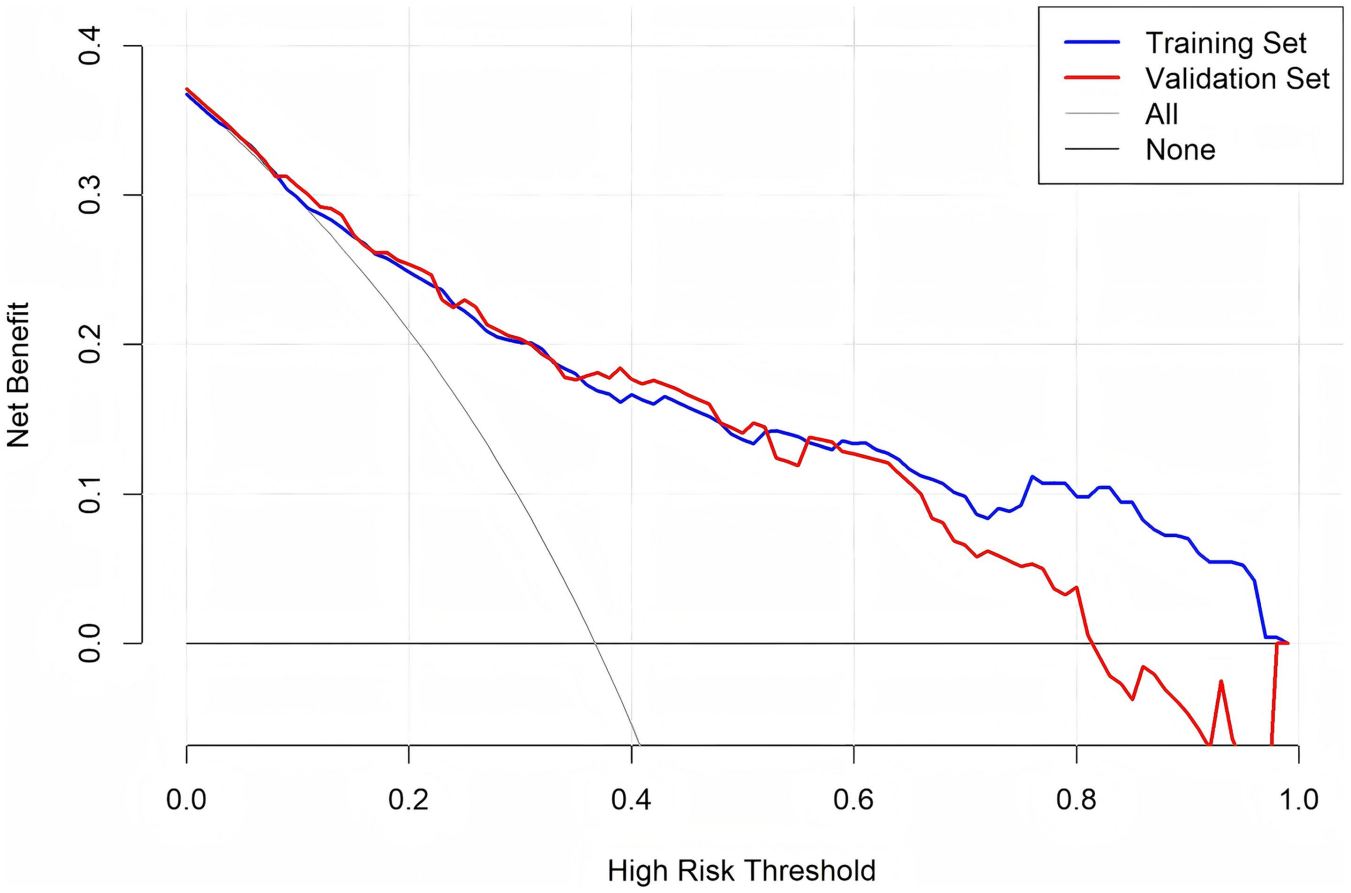
DCA of the predictive nomogram.

DCA was conducted to assess the clinical utility of the predictive model across different decision thresholds. The DCA curves for both the training and validation sets are shown in Figure 6. The model showed positive net benefit in threshold probabilities between 0.1 and 0.75, suggesting good clinical utility for risk stratification and decision-making in practice.

## 4 Discussion

In older adults with multimorbidity, the combined burden of chronic diseases and their complex interactions can significantly increase the risk of negative health outcomes (27). The CF prediction model developed in this study provides a valuable tool for improving the clinical management in this population. By expanding existing assessment frameworks, our research provides a practical approach for the early identification of CF using common clinical indicators. The model includes various physiological, psychological, and functional factors, clearly reflecting the complex nature of CF. The model aligns with the biopsychosocial framework of frailty and provides a clinically meaningful approach to risk prediction.

Among all predictors, nutritional status had the strongest influence on CF risk. Poor nutrition was strongly associated with an increased likelihood of CF, while better nutrition status was protective. Nutrition plays an important role in maintaining cognitive integrity throughout the lifespan, and significant influence on age-related cognitive decline (28). Our findings were similar to an observational study conducted in Turkey, which emphasized the importance of diet, early nutritional screening and intervention (29). Evidence also suggests nutritional support may effectively slow the mitigating cognitive decline in older adults who already exhibit signs of CF. (30) Healthcare professionals should regularly evaluate nutrition and provide personalized nutritional interventions for individuals at risk. Depression was another key factor. Data from the China Health and Retirement Longitudinal Study showed that depression increases the risk of frailty and cognitive decline (31). This may be due to the interrelation between the physiological mechanisms of depression and CF. Some studies suggest that mitochondrial dysfunction plays a major role in the pathophysiology of both depression and CF. (31) Screening for depression should to be a routine component of multimorbidity management. Integrating mental health services, including antidepressant treatment, into multimorbidity care can help reduce the psychological burden on patients.

Chronic pain was a major factor, consistent with the findings of Li et al. (32). Persistent pain can reduce physical activity, increase the risk of functional decline (33). It is also associated with nerve inflammation, higher stress hormone levels, and impaired executive function (32). Effective pain management is crucial in multimorbidity care to prevent further cognitive and physical decline. Constipation was a significant predictor of CF in multimorbid patients. Constipation, often overlooked in CF research, may influence cognitive function through the gut-brain axis (34). Changes in gut flora have been linked to neurocognitive health, indicating that gastrointestinal health affects more than just digestion (35, 36). Clinicians should actively manage constipation in older patients with multimorbidity through dietary modification, physical activity, and careful use of medications.

Polypharmacy was a notable risk factor for CF. Multimorbid patients often take multiple medications, increasing the risk of drug interactions, side effects, frailty, and medication-induced cognitive impairment (37). Regular medication reviews using deprescribing protocols can help reduce unnecessary polypharmacy. Physicians should take a personalized approach to balance disease control with minimizing risks. Compared with current drinkers, individuals who had never consumed alcohol and those who had quit drinking had significantly lower odds of CF. There has been controversy in the past regarding the impact of alcohol consumption on cognitive function and related health outcomes (38, 39). Multimorbid patients face a higher risk of alcohol-related adverse effects due to impaired liver metabolism and polypharmacy. Clinicians should adopt a personalized, risk-based approach, considering age, comorbidities, medication use, and lifestyle when advising on alcohol consumption.

Our findings emphasize the need for a multidimensional, patient-centered approach to CF prevention in older patients with multimorbidity. The developed nomogram provides a valuable tool for clinicians to personalize risk assessment and implement targeted interventions. This study has some limitations. Although external validation was conducted, the study population was geographically limited, requiring further validation in diverse population to confirm generalizability. Additionally, the model did not include biochemical markers, such as inflammatory cytokines or neurodegeneration-related proteins, which may enhance predictive value. Future research should explore the integration of these biomarkers to improve risk assessment. Furthermore, given the cross-sectional design, the associations reported here are statistical in nature, and longitudinal studies are needed to validate the predictive utility of the identified variables over time.

## 5 Conclusion

This study developed and validated a predictive model for CF in older patients with multimorbidity, which incorporated drinking, constipation, polypharmacy, chronic pain, nutrition, and depression. The model demonstrated good discriminatory ability and clinical utility, as evidenced by a high AUC, well-fitted calibration curves, and favorable DCA. The model helps identify high-risk individuals early, providing a valuable tool for guiding targeted interventions to preserve cognitive function and slow frailty progression. Future research should focus on external validation in broader populations and the inclusion of novel biomarkers to improve predictive accuracy.

## Data Availability

The raw data supporting the conclusions of this article will be made available by the authors, without undue reservation.
